# Tribo-Charging Behaviour of Inhalable Mannitol Blends with Salbutamol Sulphate

**DOI:** 10.1007/s11095-019-2612-9

**Published:** 2019-04-09

**Authors:** S. Zellnitz, J. T. Pinto, M. Brunsteiner, H. Schroettner, J. Khinast, A. Paudel

**Affiliations:** 1 Research Center Pharmaceutical Engineering GmbH, 8010 Graz, Austria; 2 Austrian Centre for Electron Microscopy and Nanoanalysis, Graz University of Technology, 8010 Graz, Austria; 3 Institute of Process and Particle Engineering, Graz University of Technology, 8010 Graz, Austria

**Keywords:** dry powder inhaler (DPI), inhalation, particle/material properties, powder blends, powder processing

## Abstract

**Purpose:**

The performance of carrier-based dry powder inhaler (DPI) formulations can be critically impacted by interfacial interactions driven by tribo-electrification. Therefore, the aim of the present work was to understand how distinct API particle characteristics affect the charging behaviour of blends intended for DPI delivery.

**Methods:**

Salbutamol sulphate (SBS) particles engineered via spray-drying and jet milling were used as model APIs. D-mannitol was selected as a model carrier. The materials were characterized concerning their different particle properties and their charge was analysed alone and in blends before and after flow over a stainless-steel pipe.

**Results:**

The spray-dried SBS (amorphous and spherical) charged positively and to a higher extent than jet milled SBS (crystalline and acicular) that charged negatively and to a lower extent. D-mannitol charged positively and to a higher extent than the APIs. All drug-excipient blends charged negatively and differences were found between the spray-dried and jet milled SBS blends at 2% and 5% drug loads.

**Conclusions:**

It was demonstrated how distinct solid-states, particle shape, size and morphology as well as different water contents of the different materials can affect tribo-charging. For their binary blends, the amount and nature of fines seem to govern inter-particle contacts critically impacting charge evolution.

**Electronic supplementary material:**

The online version of this article (10.1007/s11095-019-2612-9) contains supplementary material, which is available to authorized users.

## Introduction

Powder tribo-charging is known to greatly affect the industrial processing and performance of particulate and granular materials, including pharmaceutical solids. Issues such as un-intended particle agglomeration and adherence to container surfaces, can arise due to tribo-charging, potentially leading to feeding/dosing inaccuracy and mixing inhomogeneity (1). Moreover, powder tribo-charging can directly impact the performance of dry powder inhalers (DPIs) (2). DPIs usually contain inhalable-size active pharmaceutical ingredient (API) particles (1 – 5 μm) adhered to the surface of a larger carrier material (usually 50 – 200 μm) (3).

Tribo-electrification is a charge transfer process in the course of frictional contact and separation of two solid surfaces (4,5). This complex process depends upon several factors, such as particle/material characteristics, particle processing, as well as environmental conditions. For metals, tribo-charging can be explained by their work function (WF) values; that is the minimum energy necessary to extract an electron at the Fermi level of the solid material to the outer surface (6–8). Pharmaceutical powders are usually insulators (9) and for these the mechanism of contact charging is not yet fully understood. The concept of “effective work function” has been proposed and relates the transfer of species from high-energy states to a low energy surface during contact charging of insulators (10). Unlike metals, tribo-charging of insulators involves not only the exchange of electrons, but also of ions and material (11,12). The relative contribution of each mechanism depends upon the mode of contact and stresses generated within the materials. The resulting net charge is a consequence of the balance between the contribution of the negative and positive species (11,12). After contact, the acquired charge will dissipate (decay) at a rate specific to the material and environment (12).

Generally, pharmaceutical powders are low-conductivity materials and tend to attain electrostatic charges during powder handling and processing (9). Therefore, our work aims to understand the charging of inhalation powders and their blends during transport and processing. For the current work, our hypothesis is that blending inhalable sized API particles with different physical properties (amorphous *versus* crystalline and spherical *versus* plate like) at different concentrations with coarse carrier particles can affect the tribo-charging of the blends in a diverse way. For that, amorphous and crystalline salbutamol sulphate particles with distinct properties were chosen as model API fines (13). As a model carrier, we used a mannitol as it is rapidly evolving as an alternative inhalation carrier to lactose (for typical instability problems like drug-excipient Maillard reactions) (14). Coarse mannitol was mixed to generate blends with fine API at different loadings. Tribo-charging of these powders during flow over a stainless steel tube was investigated in order to obtain relevant information of their behavior during unit operations such as feeding, transport, blending and capsule filling, etc. The results of relative charging in the blends were compared to the individual materials and interpreted using the different physical properties of the individual components as well as of the blends.

## Materials and Methods

### Materials

Salbutamol sulphate (SBS) was purchased from Selectchemie (Zurich, Switzerland) and used as a model drug. D-mannitol (MAN), Pearlitol® 160 C from Roquette (Lestrem, France) was used as a coarse model carrier material.

### Particle Engineering

Crystalline fine salbutamol sulphate was produced by milling. Jet milling was performed using a Spiral air Jet Mill 50 AS, (Hosokawa Alpine AG, Augsburg, Germany) at an air injection pressure of 6 bar and a milling pressure of 3 bar. Powder feeding was performed manually (15).

A Büchi Nano spray-dryer B-90 (Büchi, Flawil, Switzerland) was used to produce fine amorphous particles of salbutamol sulphate by drying its aqueous solution (7.5 wt%) at a feed rate of 0.3 mL/min and an air flow rate of 110 L/min. A piezoelectric vibrating mesh of 7 μm, an inlet and outlet temperatures of 120°C and 49°C, respectively were found appropriate to generate inhalable particles (16).

All samples were stored under ambient conditions (28 ± 3% RH, 22 ± 2°C) in sealed glass vials prior to analysis and blend preparation.

### Material Characterization

#### Particle Size Characterisation

The volumetric particle size distribution (PSD) of the samples was determined using laser diffraction (HELOS/KR, Sympatec GmbH, Clausthal-Zellerfeld, Germany) coupled with a dry dispersing system (Rodos/L, Sympatec) and a vibrating chute (Vibri, Sympatec). The PSD data were generated using a primary dispersion pressure of 2.0 and 0.5 bar for the micronized SBS and coarse MAN, respectively. The volume particle size (D10, D50 and D90) and SPAN were determined and used to describe the PSD of the samples. D10, D50 and D90 describe the diameter corresponding to 10%, 50% and 90% of the cumulative undersize of the obtained volume distribution, respectively. These were used to calculate the width of the distribution, expressed as the SPAN = (D90-D10)/D50. Measurements were made in triplicate, with an average sampling time of 30 s and data evaluation was performed using the software Windox 5.6.0.0 (Sympatec).

#### Scanning Electron Microscopy (SEM)

The surface morphology of MAN as well as jet milled and spray-dried SBS particles was observed using scanning electron microscopy (SEM) operated at 5 kV (Zeiss Ultra 55, Zeiss, Oberkochen, Germany). Prior to analysis, all samples were gold palladium sputtered.

#### Wide Angle X-Ray Scattering (WAXS)

The solid-state of the selected materials was characterized via wide angle X-ray scattering (WAXS). The samples were placed inside 2 mm glass capillaries and analysed under constant rotation (9 rpm/min) using a point-focusing camera system S3-Micro (Bruker AXS GmbH, Germany). Measurements (*n* = 2) were carried out in the range between 17.2°-27.9° 2θ at room temperature (22 ± 2°C) during 600 s.

#### Determination of Unbound Water in the Powder Samples

The unbound water content of the samples was experimentally determined by estimating the weight loss of the powder sample stored under vacuum (VacPrep 061, Micromeritics, USA) at room temperature (22 ± 2°C) for 48 h.

#### Calculation of Work Function (WF) Values of Individual Materials

The WFs of the materials were estimated based on the ionization potential and the energy band gap at the centre of the HOMO (highest occupied molecular orbital) and LUMO (lowest unoccupied molecular orbital) (17) using Eq. (1):

1 $$ \mathrm{WF}\approx \mathrm{IE}-0.5\times {\mathrm{E}}_{\mathrm{HOMO}}-{\mathrm{E}}_{\mathrm{LUMO}} $$

where, IE is the ionisation energy and E_HOMO_ and E_LUMO_ the energy of the HOMO and LUMO states, respectively. The IE, E_HOMO_ and E_LUMO_ were calculated using an open source software (MOPAC2016, Stewart Computational Chemistry, USA) at the PM7 level of the semi-empirical NDDO (neglect of diatomic differential overlap) theory.

### Preparation of Adhesive Mixtures

Electrostatic charging during the blending of D-mannitol has been extensively studied by Karner *et al*., and blending parameters in the present work were chosen accordingly (18). Mixtures of coarse MAN with either spray-dried and jet milled SBS at two different drug loads (2 and 5 wt%) were prepared in a tumble blender TC2 (Willy A. Bachofen Maschinenfabrik, Muttenz, Switzerland) (28 ± 2% RH, 21 ± 0°C). 0.2 g of API fines (spray dried or milled) and 9.8 g of mannitol (2 wt%) or 0.5 g of fine material and 9.5 g of mannitol (5 wt%) were layered in stainless steel vessels (filling volume about 40%) using the sandwich method. Before blending, all SBS samples were sieved through a 400 μm mesh to break large agglomerates and mixing was performed for 60 min at 60 rpm. As a control, MAN was processed alone under the same conditions and is referred hereafter as blended MAN. A 75 ml closable stainless steel vessel with an inner diameter of 49 mm and inner height of 40 mm was used as a powder container for mixing.

### Charge Measurements

**Fig. 1:**
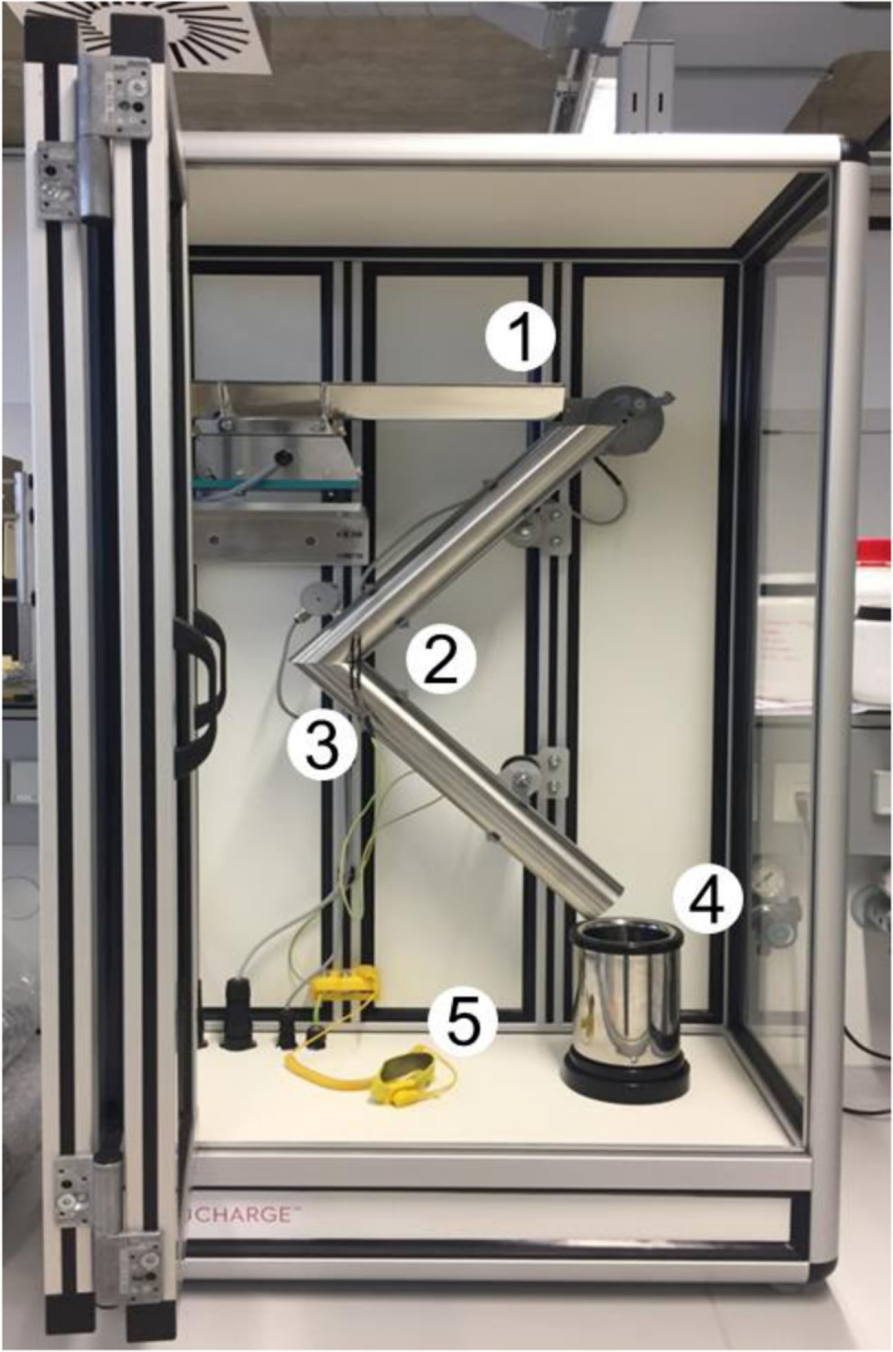
Experimental setup for charge measurements; (1) vibrating chute, (2) stainless steel v-tubing, (3) grounding cable, (4) Faraday cup and (5) antistatic bracelet.

In the present study, all tribo-charging measurements were carried out using the Granucharge™ (Granutools, Awans, Belgium) apparatus shown in Fig. 1. The charge measurements were performed immediately after blending at the following RH and temperature conditions 28 ± 2% RH and 21 ± 0°C, respectively. In order to minimize the impact of the environment, the sampling time was limited to a maximum of 5 min (for *n* = 3). First, the initial charge of the powders (q_0_) was recorded by dispensing the sample directly into a Faraday cup using a stainless steel spoon. The powder was then transferred to a vibrating chute from where it was delivered to a stainless steel V-tube to fall again into the Faraday cup in order to measure its total charge (q_1_). Accordingly, the net charge induced by tribo-electrification is the difference between q_1_ and q_0_*,* (q_1_-q_0_) (see details in supplementary material, Table A1 and A2). The charge per unit of powder mass (Q/m, nC/g) was automatically determined as the ratio between the measured charge (Q) and sample weight (m). Each analysis was done with about 3 g of powder. Between each replicate measurement of the same sample, the remaining powder in the tubes and Faraday cup were removed with paper towels. To normalize the contribution of particle size and surface area, the charge per specific surface area unit (Q/A, nC/m^2^) was determined as follows (Eq. 2).

2 $$ \frac{\mathrm{Q}}{\mathrm{A}}=\frac{\left(\frac{\mathrm{Q}}{\mathrm{m}}\right)\times \mathrm{powder}\ \mathrm{mass}}{\mathrm{SSA}} $$

Specific surface area (SSA) values for the raw materials were taken from gas adsorption measurements described in our previous works (13,19). The SSA of the blends was assumed to be the sum of the weighted SSA of the SBS and MAN particles divided by the total mass of the blends (Table I). Since our aim was to compare relative values, the SSA of the blends were theoretically calculated with the assumption that the potential deviation from the experimental SSA will not have an overall critical effect on the systematic normalization of the results.

**Table I:** Specific Surface Area (SSA) of the Raw Materials and Their Respective Blends

Fine material	SSA materials* [m^2^/g]	SSA 2 wt% blend** [m^2^/g]	SSA 5 wt% blend** [m^2^/g]
SBS SD	1.570	0.245	0.286
SBS JM	11.040	0.434	0.759
MAN	0.218	–	–

*SSA taken from (13,19)

**SSA = (Area fines  × wt% fines + Area carrier × wt% carrier)/10

#### Statistical Analysis

Differences in the tribo-charging behavior of the individual materials and the drug-carrier respective blends were statistically evaluated using a two-way analysis of variance (ANOVA). GraphPad Prism 7 (GraphPad Software, USA) was used to carry out the evaluations.

## Results

### Physical Characterization of the Raw Materials

#### Micromeritics and Morphological Properties

**Fig. 2:**
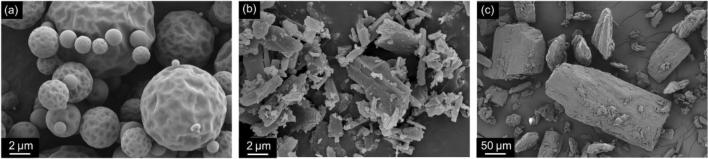
SEM images of (**a**) SBS SD, (**b**) SBS JM at an image width of 22.87 μm and (**c**) MAN at an image width of 571.5 μm.

SEM images in Fig. 2a and b show distinctive morphologies for the SBS prepared by spray-drying (SBS SD) and jet milling (SBS JM). SBS SD particles were spherical, whereas SBS JM particles were irregular shaped. The particle size of both SBS SD and SBS JM samples were in the inhalable size range (< 5 μm). When compared to their SBS JM counterparts, the SBS SD particles showed a slightly larger volume mean diameter (D50), as well as narrower size distribution (Table II). The surface of the SBS SD particles was smooth and indented for some larger particles (Fig. 2a). In contrast, jet milling (SBS JM) originated very irregular flake-like particles (Fig. 2b). Additionally, it was observed that MAN particles showed a similar size to that of other carriers used in inhalation, i.e. Lactohale 120 M (DFE Pharma, Goch, Germany) and Inhalation 80 M (Sheffield Bio-Science, Norwich, USA) (20). Moreover, SEM inspection revealed that the excipient powder was composed of cubic irregular shaped particles with a relatively rough surface (Fig. 2c).

**Table II:** Volume Mean Particle Size Distribution and SPAN of the Raw Materials (*n* = 3 ± SD)

Materials	D10 [μm]	D50 [μm]	D90 [μm]	SPAN*
SBS SD	0.59 ± 0.02	3.68 ± 0.05	7.57 ± 0.20	1.90 ± 0.03
SBS JM	0.50 ± 0.02	1.89 ± 0.15	6.01 ± 0.84	2.90 ± 0.21
MAN	18.23 ± 0.38	92.18 ± 1.08	212.11 ± 2.10	2.10 ± 0.00

*SPAN = (D90-D10)/D50

#### Solid-State Properties

**Fig. 3:**
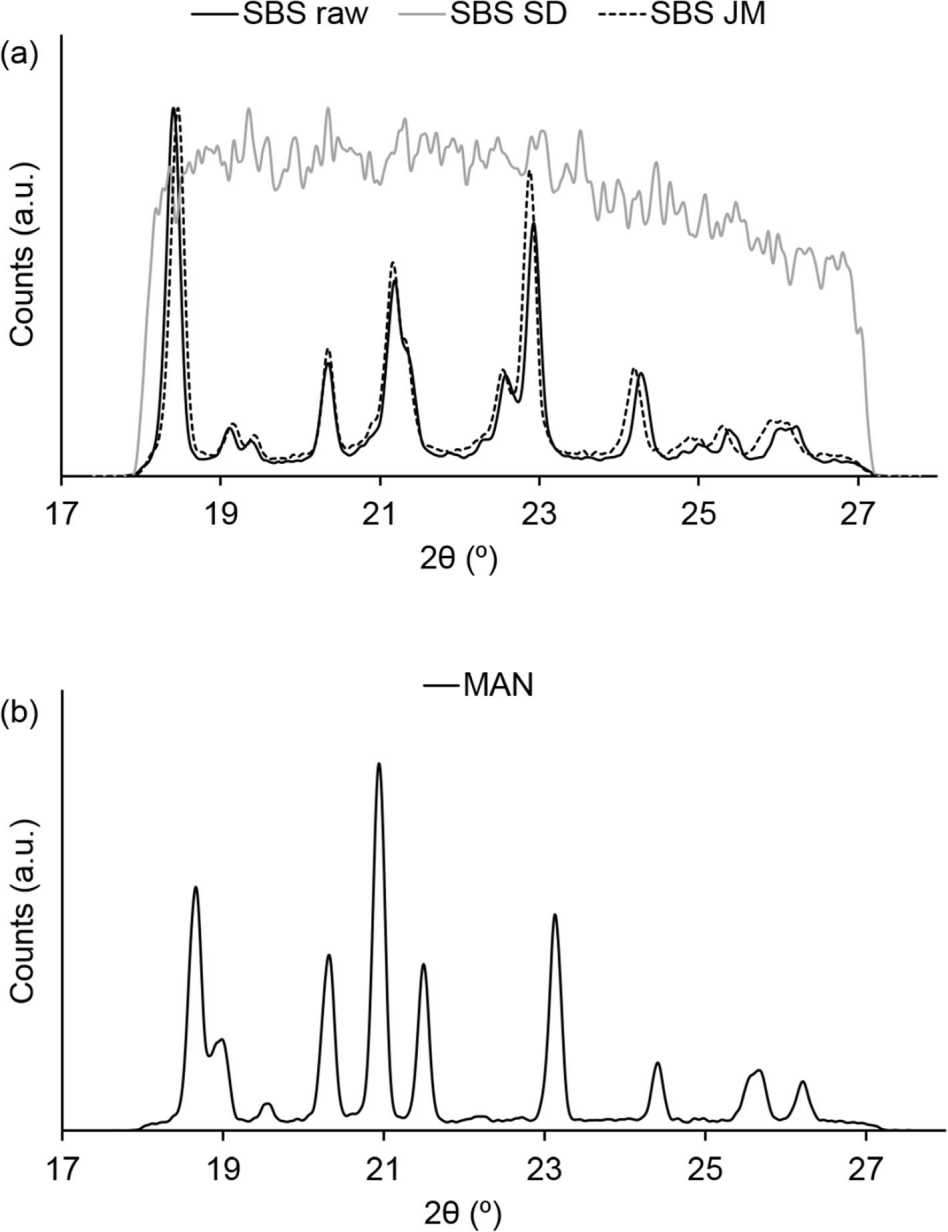
X-ray analysis of (**a**) the distinct engineered SBS particles and (**b**) MAN.

As reported in our previous studies, spray-drying of salbutamol sulphate led to the appearance of an amorphous phase and jet milling to a crystalline one (13). This was confirmed by the presence of characteristics Bragg peaks and a diffuse halo in the WAXS patterns of jet milled and spray-dried particles, respectively (Fig. 3a). The similar peak patterns of the raw and jet milled powders reflected the pre-existing crystal form of SBS. Analysis of the coarse MAN particles (Fig. 3b) showed that the sample is predominantly composed of the β polymorph, as evidenced by the peak at 23.1° (21). However, an inversion of the intensity ratio between the peak at 20.9°C and 23.1°C 2θ was observed. This implied the potential presence of trace amounts of other polymorphic phases of MAN in the sample.

#### Surface Properties

Gravimetric data on unbound water content showed a significant loss of weight for the spray-dried samples corresponding to a water percentage of 3.2 wt%. On the other hand, the milled material showed only a negligible loss on drying, corresponding to a water content of 0.18 wt%. This is in agreement with work from Chiou *et al*. who reported that crystalline SBS particles generated via high-gravity controlled precipitation are much less hygroscopic compared to amorphous SBS particles generated via spray-drying (22). Compared to other sugars, mannitol is known to be less hygroscopic. This is reflected by the loss on drying results for mannitol, corresponding to a water content of 0.02 wt%.

The applicability of WF values to organic materials as a qualitative comparison parameter was explored in this work. Likewise, a semi-empirical methodology was applied to calculate the WF of SBS and MAN and the results are presented in Table III. SBS shows a propensity to accept electrons when in contact with stainless steel, which is in accordance with their observed charging tendency. In contrast MAN has shown to donate electrons when in contact with stainless steel.

**Table III:** Work Function of the Contacting Surface and Raw Materials

Material	Work function (eV)
SBS	7.7
MAN	4.3
Stainless steel	4.4* / 5.1**

*value from (23), **value from (24)

### Tribo-Charging of the Raw Materials

In the following chapters we chose the terms higher/lower charge or tribo-charging for a charge either more negative (−) or more positive (+) compared to the reference point.

As most of the formulations comprise of the carrier (95 wt% and 98 wt%), MAN was blended alone (or rather tumbled in the blender identical to that of true blending) and results were compared with the untreated sample.

**Fig. 4:**
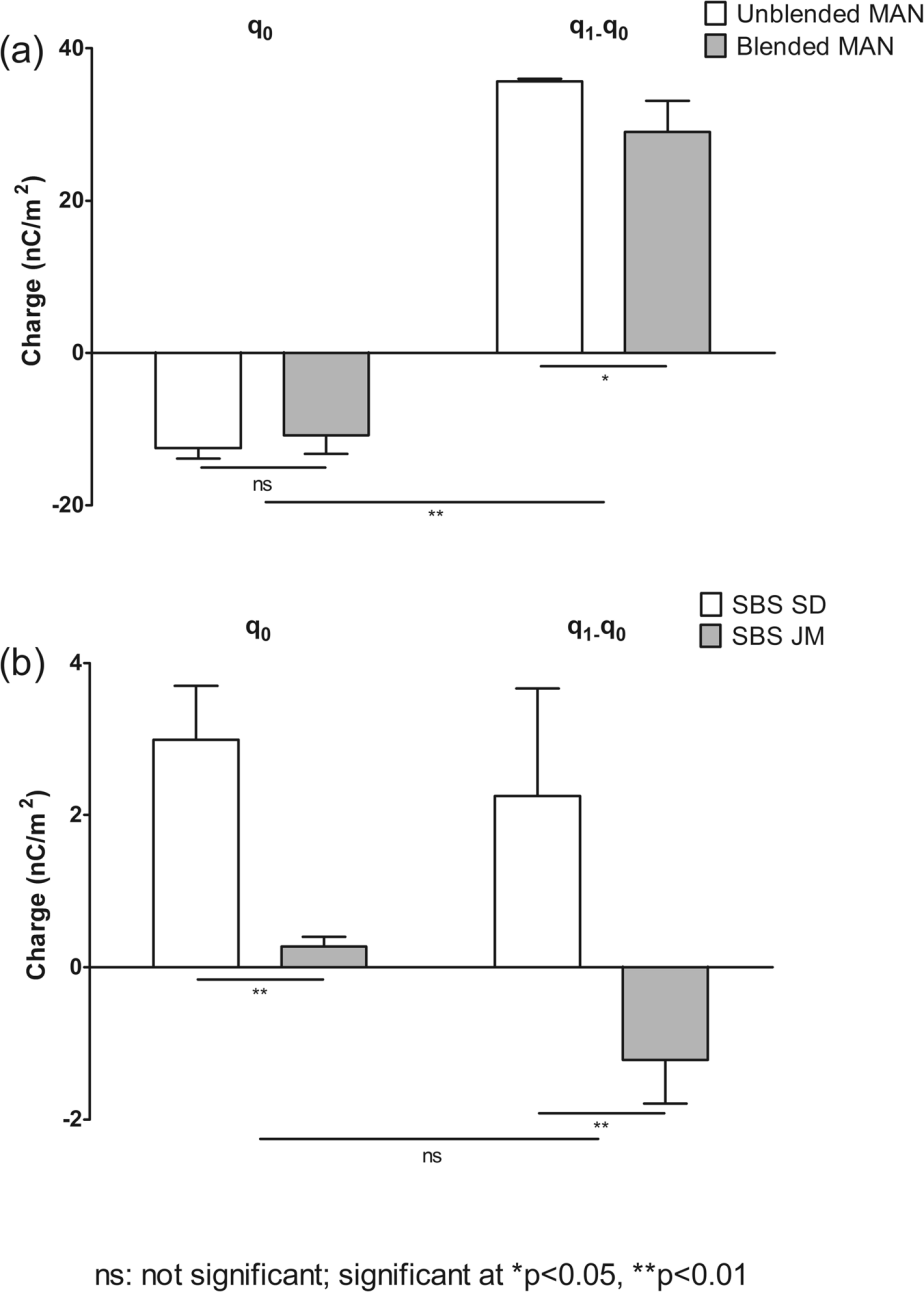
Raw materials initial charge – q_0_ and charge after flow – q_1_-q_0._ (**a**) MAN and (**b**) the distinct engineered SBS particles (n = 3 ± SD). Statistical analysis was carried out using a two-way ANOVA.

Figure 4a shows that both untreated and the blended mannitol charged negatively and to the same extent before contact with stainless steel (−12.54 ± 1.33 nC/m^2^ and − 10.83 ± 2.44 nC/m^2^, respectively). After flow over steel, an overall positive density of charge was found (35.67 ± 0.31 nC/m^2^ and 29.06 ± 4.08 nC/m^2^, respectively), with the blended MAN showing a lower tendency to charge when in contact with stainless steel. It was found that flow led to a significant change in MAN charge, as revealed by the notable different values found before (q_0_) and after flow (q_1_-q_0_) of the powders over the stainless steel pipe.

Results in Fig. 4b show that SBS powders have a positive charge before flow over stainless steel (2.99 ± 0.71 nC/m^2^ and 0.28 ± 0.13 nC/m^2^ for SBS SD and SBS JM, respectively), with SBS SD charging significantly more than its SBS JM counterpart when in contact with air.

After flow over stainless steel, the distinctly engineered SBS particles, showed contrasting charging tendencies. SBS SD charged positively (2.25 ± 1.42 nC/m^2^) and SBS JM negatively (−1.21 ± 0.57 nC/m^2^). Interestingly, flow of the powder over the stainless steel pipe had little influence on the charging tendency of the materials. Overall, no statistical difference was found when comparing the values before (q_0_) and after (q_1_-q_0_) flow even though there was an inversion of charge for SBS JM.

### Tribo-Charging of the Blends

To evaluate the impact of SBS particle properties on the charging of the coarse carrier based formulations, blends with 2% (wt%) and 5% (wt%) SBS loads were prepared. After blending, the total amount of the mixtures was passed through the stainless steel V-tube in order to understand if the charging behaviour of the carrier particles is affected by mixing with SBS particles.

**Fig. 5:**
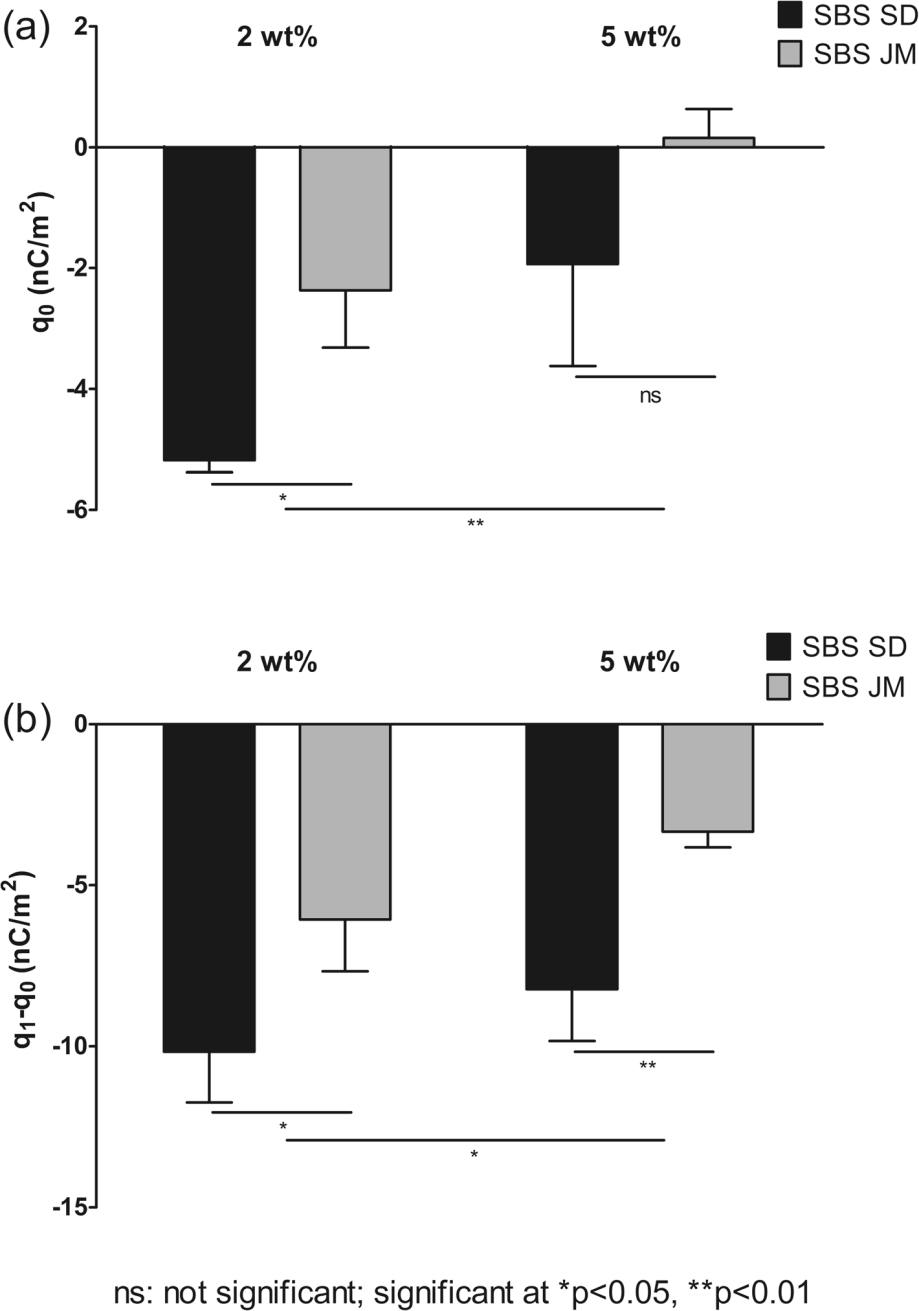
Initial charge – q_0_ (**a**) and charge after flow – q_1_-q_0_ (**b**) of the blends of MAN with the distinct engineered SBS particles (n = 3 ± SD). Statistical analysis was carried out using a two-way ANOVA.

Interestingly, a significant difference in the charging tendency could be observed between the carrier alone and its adhesive mixtures. All MAN blends charged negatively and to a lower extent than the MAN carrier blended alone (Fig. 5).

Before contact with stainless steel (q_0_), the MAN-SBS SD 2% and MAN-SBS JM 2% blends had a charge of −5.18 ± 0.20 nC/m^2^ and − 2.37 ± 0.95 nC/m^2^, respectively (Fig. 5a). Flow resulted in a higher acquisition of charge (q_1_-q_0_) for the SBS SD blends than for its SBS JM counterparts, i.e., −10.17 ± 1.57 nC/m^2^ and − 8.23 ± 1.61 nC/m^2^, respectively (Fig. 5b). At higher drug loads (5 wt%), a slightly lower charge was found before and after flow. Before contact with stainless steel, the SBS SD and the charge of SBS JM blends was −1.94 ± 1.68 nC/m^2^ and 0.15 ± 1.48 nC/m^2^, respectively. Flow over steel led to charge values of −6.06 ± 1.78 nC/m^2^ and − 3.34 ± 0.49 nC/m^2^ for the SBS SD and SBS JM blends, respectively. Overall, the blends of SBS SD and SBS JM showed to charge significantly different (*p* < 0.05), with the exception of the charge before flow of the blends containing 5 wt% SBS (*p* > 0.05). Overall, increasing the load of SBS from 2 wt% to 5 wt% showed to have a significant impact on the charging behaviour of the blends both before (q_0_) and after flow (q_1_-q_0_).

## Discussion

In the following section, we will discuss how the particle properties and combinations thereof could qualitatively relate to the observed tribo-charging results.

### Evolution of Charge in Starting Materials

#### Material Properties

Since tribo-charging is a surface phenomenon, species exchange can be affected by differences in the physical and chemical properties of molecules at the surface of materials (8). Therefore, the WF, an important surface parameter describing the electronic structure of interfaces, was calculated for SBS and MAN. The ranking of the WF values in Table III suggested that SBS has a stronger attraction for electrons than steel, while steel attracts electrons more than MAN. Thus, after flow over steel, it would be expected that SBS charges negatively and MAN positively. For the crystalline materials (SBS JM and MAN) after contact with stainless steel experimental results showed a similar charge tendency to the one predicted (SBS JM charged negatively and MAN positively). However, attesting to the limited applicability of the calculated WF was the fact that the same material, i.e., SBS SD, showed an inversion of charge when powders with different particle properties were tested.

Concerning the charging of crystalline and amorphous SBS powders after aerosolization, Wong *et al*. reported similar findings with the amorphous SBS charging positively and crystalline SBS negatively (24). The authors explained the contrasting tendencies of charge with the distinct atomic surface arrangements of the molecules at the crystalline and amorphous phases. However, they pointed out the possible contribution of other factors as well (i.e., water content and surface roughness). Moreover, and similarly to our result, the amorphous SBS also charged to a higher extent than its crystalline counterpart.

#### Mode of Contact Effect on Tribo-Charging

Charging will, evidently, depend upon how two materials contact with each other (11). Particularly, particle shape can influence the mode of contact between two surfaces. Generally, spherical particles tend to roll and gather higher charges than flat particles that tend to slide in a quasi-fixed position (25). However, under certain conditions described by Ireland (dependent on their roundness ratio and friction coefficient), flat particles can begin to tumble. Consequently, it is possible that more flatted-like irregular particles are prone to undergo violent tumbling and bounce and thus attain a higher magnitude of charge than spherical ones. This might explain why the milled SBS and MAN showed to acquire more charge after flow than the SBS SD that almost showed no difference in relation to its initial charge (q_0_). Similar findings were obtained by Kaialy *et al*., who reported a considerably lower net charge for spherical MAN particles compared to irregularly-shaped MAN ones (26). However, further investigations are needed in order to elucidate how different shaped particles flow over the used stainless steel V-tube.

Besides shape, it is well-known that the nature of contacts between two surfaces is dependent on the mechanical properties of their contacting surfaces. Owing to differences in their molecular order, amorphous and crystalline phases of the same material present different mechanical properties (27). Moreover, small particles typically have a higher specific comminution energy due to a different defect structure than larger particles. Thus, smaller particles are harder than their larger counterparts. Although, mechanical characteristics were not directly investigated in this work, we note that the different particulate properties considered might have led to distinct mechanical moduli that could have affected the mode of contact.

#### Effect of Unbound Water on Tribo-Charging

Water molecules surrounding solid particles have been proposed to be the driving force in the tribo-charging of insulator materials (28). Although the exact role of water is still unclear, it seems that it is related to the quantity of water molecules present on a surface of a solid. For instance, when few water molecules are adsorbed at the surface, charge transfer can occur due to several proposed mechanisms, leading to tribo-electrification of the material (29,30). By contrast, water can also lead to charge mitigation in cases when materials adsorb enough molecules to form an aqueous layer that is further able to increase conductivity (28,31). Thus, depending on the type of material, different relative humidities can affect tribo-charging distinctly (29,30,32).

Relating to our work and considering the high water content of the SBS SD powders, this might have affected tribo-charging. It is hypothesized that in contact with air, before flow, the excess water mediated tribo-charging. This explains why a higher charge was found for the SBS SD powders in relation to its SBS JM counterpart. However, during flow the charges generated by contact with stainless steel were more easily conducted and dissipated by the amount of water present in the powder. This poses another explanation why almost no difference was seen in the charging of SBS SD before and after flow. For instance, it is shown in literature for SBS that an increase of the water content of SBS powder from about 0.0 to 0.2 wt% is enough to lower the time required for the charge dissipation after corona charging from ca. 900 to 90 min (33).

### Charge Evolution in Binary Blends

Charge evolution of binary blends with bimodal particle size distribution has been the topic of numerous publications. Although similar observations to our work are found for binary blends, the corresponding interpretations and explanations are not clearly stated so far (34–37). Taking into account the literature about blends of the same material with a bimodal particle size distribution, it is known that inter-particle contacts result in smaller particles tendentiously acquiring negative charge and coarse ones positive (38,39). Moreover, as charge density increases, coarse-fine contacts will dominate (instead of same sized particle contacts) (34) and a charging transfer continuum is produced resulting in an average net balance that lies somewhere between the overall charge of single particles (35). Although blends in the current study are of two different materials, we hypothesize that a similar phenomenon contributes to a part of the results. As the number of particles in the mixtures increases (from 2 to 5 wt% fine content) a higher occurrence of contacts will result in a higher charge density and fine-coarse interaction. Under these circumstances, charge mitigation might occur due to (1) increased charge segregation and/or (2) buffering effects. The first scenario is proposed based on the work of Forward *et al*. for charging of bimodal blends of the same material. In this work, the authors have shown that for a higher ratio of the diameter of larger-to-smaller particle, a fraction of finer material of 7 wt% is enough to cause the largest charge segregation between particles (39). Thus, it is possible that a similar effect occurs within the tested blends leading to a new charge continuum resulting in an overall net charge balance different from the one found for the coarse particles that dominate the blend (98 and 95 wt%). For the second scenario, it is suggested that inter-particle contact times will be ruled by the surface physicochemistry of the particles. Accordingly, particles that are more prone to attachment to a certain solid surface, due to their physicochemical properties, will remain attached and dominate contacts during tribo-charging. From our previous works, we know that SBS SD is more cohesive than SBS JM. The latter, in turn, showed a higher tendency to attach to the surface of lactose monohydrate and D-mannitol (13,40). As such, SBS JM surface physicochemical interactions with MAN will be more favourable, promoting the adherence of fine particles to the coarse MAN carrier surfaces and contacts will be dominated by the fine particles and the blends will charge to a lower extent (fines showed to charge to a lower extent that the coarse material).

Additionally, it is also proposed that in the case of SBS SD, a smaller charge mitigation was observed because SBS SD has less propensity to gain charge (during sliding than the SBS JM) and has a larger size (less particle contacts). So, for the same blend mass, more fine material would be needed to cause the same charge induction found for the SBS JM blends.

Although, we hypothesize that coarse-fine particle contacts dominate over same particle and particle-wall contacts, the contribution of the latter cannot be entirely excluded from the overall net charge balance found.

As mixing was also carried out in stainless steel vessels (prior to tribo-charging test), the mechanisms described above and the forces to induce particle-particle contacts could have affected tribo-charging of the blends either (1) already during blending or/and (2) in-situ during dispersing and sliding.

## Conclusion

The present work compared and contrasted the charging behaviour of pure pharmaceutical solids of different size, shape and solid state and investigated the tribo-charging of binary powder blends (coarse and fine particle blends). Distinct surface chemistry arising from the solid-states of the material, different contact areas due to particle properties like shape, size and morphology, as well as variable water contents have shown to critically impact the polarity and magnitude of tribo-charging. It was demonstrated that amorphous, spherical shaped SBS particles with a high water content (generated via spray-drying) attained positive charge of higher magnitude. In contrast, milled fine particles with irregular shape and lower water content tend to charge negatively and to a lower extent. For the crystalline powders, the theoretically calculated WFs were able to provide the rank order of the sign of charge in line with the experimental values. The charging tendencies of the carrier material seem to be less relevant when powder blends are prepared with typical fine (API) loads of 2 to 5 wt%. All blends charged negatively and to a lower extent than the coarse MAN alone. Evolution of charge seemed to be dominated by the nature of fines in relation to coarse particles and their concentrations contributing to inter-particle contacts. We emphasize here that the investigation of charging propensity of an extensive set of diverse particulate materials and their control blends is certainly required to substantiate our conclusions. Overall, these data also clearly reflect the need of further work towards understanding the tribo-charging behaviour of diverse pharmaceutical solids (ordered *versus* disordered packing, free *versus* bound water, salt *versus* free acid/base form etc.).

## Electronic supplementary material

ESM 1 (DOCX 29.4 kb)

## Abbreviations

API: Active pharmaceutical ingredient
APSD: Aerodynamic particle-size distribution
COPD: Chronical obstructive pulmonary disease
DPI: Dry powder inhaler
SBS JM: Jet milled salbutamol sulphate
MMAD: Mass median aerodynamic diameter
MAN: Mannitol
NGI: Next generation impactor
PSD: Particle size distribution
SEM: Scanning-electron microscopy
SBS: Salbutamol sulphate
SBS SD: Spray-dried salbutamol sulphate
WF: Work function
