# Supplementary material for: Tribo-Charging Behaviour of Inhalable Mannitol Blends with Salbutamol Sulphate
Source: Pharm Res. 2019 Apr 9;36(6):80. doi: 10.1007/s11095-019-2612-9 (PMC6456482; doi:10.1007/s11095-019-2612-9)
Supplement: Supplementary file 1 — (DOCX 29.4 kb) [file 11095_2019_2612_MOESM1_ESM.docx]

**Supplementary Material**

**Table A1:** Tribo-charging results for the raw materials

| Material | q_0_  [nC/m^2^] | q_1_  [nC/m^2^] | q_1_ - q_0_  [nC/m^2^] |
| --- | --- | --- | --- |
| SBS SD | 1.60 ± 0.38 | 2.814 ± 0.78 | 1.21 ± 0.76 |
| SBS JM | 0.80 ± 0.37 | -2.74 ± 1.37 | -3.54 ± 1.67 |
| MAN | -23.30 ± 2.47 | 43.56 ± 2.36 | 66.86 ± 0.60 |

**Table A2:** Tribo-charging results for the blends

| Blend | q_0_  [nC/m^2^] | q_1_  [nC/m^2^] | q_1_ - q_0_  [nC/m^2^] |
| --- | --- | --- | --- |
| MAN + SBS SD 2% | -7.32 ± 0. 28 | -21.68 ± 2.42 | -14.36 ± +2.22 |
| MAN + SBS SD 5% | -2.15 ± 1.86 | -11.28 ± 1.25 | -9.13 ± 1.78 |
| MAN + SBS JM 2% | -4.84 ± 3.10 | -19.19 ± 1.80 | -14.35 ± 4.53 |
| MAN + SBS JM 5% | 0.39 ± 1.21 | -8.05 ± 0.29 | -8.44 ± 1.23 |
